# PP-DDP: a privacy-preserving outsourcing framework for solving the double digest problem

**DOI:** 10.1186/s12859-023-05157-8

**Published:** 2023-01-31

**Authors:** Jingwen Suo, Lize Gu, Xingyu Yan, Sijia Yang, Xiaoya Hu, Licheng Wang

**Affiliations:** 1 State Key Laboratory of Networking and Switching Technology, Beijing University of Posts and Telecommunications, Beijing, China; 2 School of Cyberspace Science and Technology, Beijing Institute of Technology, Beijing, China

**Keywords:** Double digest problem, Privacy-preserving, Outsourcing computation, Order-preserving homomorphic index scheme, Quantum inspired genetic algorithm

## Abstract

**Background:**

As one of the fundamental problems in bioinformatics, the double digest problem (DDP) focuses on reordering genetic fragments in a proper sequence. Although many algorithms for dealing with the DDP problem were proposed during the past decades, it is believed that solving DDP is still very time-consuming work due to the strongly NP-completeness of DDP. However, none of these algorithms consider the privacy issue of the DDP data that contains critical business interests and is collected with days or even months of gel-electrophoresis experiments. Thus, the DDP data owners are reluctant to deploy the task of solving DDP over cloud.

**Results:**

Our main motivation in this paper is to design a secure outsourcing computation framework for solving the DDP problem. We at first propose a privacy-preserving outsourcing framework for handling the DDP problem by using a cloud server; Then, to enable the cloud server to solve the DDP instances over ciphertexts, an order-preserving homomorphic index scheme (OPHI) is tailored from an order-preserving encryption scheme published at CCS 2012; And finally, our previous work on solving DDP problem, a quantum inspired genetic algorithm (QIGA), is merged into our outsourcing framework, with the supporting of the proposed OPHI scheme. Moreover, after the execution of QIGA at the cloud server side, the optimal solution, i.e. two mapping sequences, would be transferred *publicly* to the data owner. Security analysis shows that from these sequences, none can learn any information about the original DDP data. Performance analysis shows that the communication cost and the computational workload for both the client side and the server side are reasonable. In particular, our experiments show that PP-DDP can find optional solutions with a high success rate towards typical test DDP instances and random DDP instances, and PP-DDP takes less running time than DDmap, SK05 and GM12, while keeping the privacy of the original DDP data.

**Conclusion:**

The proposed outsourcing framework, PP-DDP, is secure and effective for solving the DDP problem.

## Background

With the rapid development of human genomics technology, both life science and computing technology have profoundly changed. High-throughput next-generation sequencing (NGS) technology, single-molecule sequencing technology and other technologies have emerged in this scientific revolution [1]. The next-generation sequencer generated a large number of sequence fragments, which are called read segments [2]. As these segments are too short to carry enough valid information, it is important to reorder and compare these short read segments to the reference genome to obtain genetic information. Being a fundamental problem in NGS technology, the researches on the double digest problem (DDP) aim to rebuild the target DNA sequence by recombining these fragments together in a proper order [8]. It uses two kinds of enzymes, enzyme $\alpha$ and enzyme $\beta$ to cut a long target DNA sequence into short fragments. There are three different cutting ways: cutting by $\alpha$, cutting by $\beta$, and cutting simultaneously by $\alpha$ and $\beta$. Then, by using the gel-electrophoresis experiments for each case, three sets *A*, *B* and *C* which contain the length of these fragments can be obtained. As the foundation of NGS technology, the double digest problem is of great significance.

### Existing algorithms for the double digest problem

In the 1970 s, Smith et al. [3, 4] reconstructed the physical map of DNA, then more researchers began to follow the interest in the DDP problem and many algorithms have been proposed to solve the problem. Schmitt and Waterman [5] introduced equivalence classes on DDP solution sets, solved the DDP problem by using cassette transformations and posed an open problem fully characterizing equivalent physical mappings. Pevzner [6] generalizes the cassette transformations and characterizes equivalence classes of pairwise maps under these transformations. He proved that the solution of DDP is closely related to alternating Eulerian cycles in colored graphs. Wu and Zhang [7] proposed using integer programming techniques to solve the DDP problem and increase the scale of problem-solving. However, it can’t hand off the errors in the experimental data. Later, some genetic algorithms were proposed to solve the DDP problem. However, the genetic algorithm proposed by Sur-Kolay et al. [8] could only handle errorless data, Ganjtabesh [9] improved Sur-Kolay’s algorithm, and extended it to erroneous data. In 2019, Wang et al. [10] modeled the DDP problem by using vectors and developed a MATLAB package with six genetic operators for solving the DDP problem. To improve the efficiency of the genetic algorithm, a quantum genetic algorithm combining quantum computing and the genetic algorithm has been proposed to solve the DDP problem by Suo et al. [11]. Among these algorithms, quantum inspired genetic algorithm outperform the others.

However, in the NGS technology, it is practically impossible for personal computers to support a sequencer measuring millions of short DNA sequence fragments. Thus, it is interesting to design a outsource framework for solving the DDP problem by using the capability of the cloud platforms. However, outsourcing the task of solving DDP to third party cloud servers might put sensitive genetic data at severe risk. To prevent privacy leaking, the lengths of the DNA fragments produced in the gel-electrophoresis experiments of DDP have to be protected as they are unique and unalterable. Meanwhile, the personal genetic information conveyed by them is of commercial value. Therefore, a privacy-preserving outsourcing framework for solving DDP is expected.

### Privacy-preserving outsourcing frameworks

There is a lot of research on secure outsourcing computation. The most widely used methods for privacy protection include homomorphic encryption, secure multi-party computation, cryptographic hash function and so on [12]. In 2016, Wang et al. [13] proposed the HEALER framework, homomorphic computing is used to safely assess genetic associations with rare variants of the phenotype without the involvement of the data owner, and a compression technique is proposed to reduce genome data size and communication cost. Ghasemi et al. [14] presented a data outsourcing model. The privacy of the genomic database is guaranteed by encrypting each record of the linear operation using the Paillier encryption scheme. But it only encrypts a small fraction of the DNA sequences in the entire dataset, so it lacks privacy. In 2020, Liu et al. [15] proposed LightCom, a framework for outsourcing to the cloud. Users only need to use one server to realize the safe storage and processing of data. Specifically for LightCom, a security framework is proposed, under which the server is fully equipped with multiple trusted processing units, under which side-channel attacks may occur. In 2021, Kim et al. [16] developed secure genotype imputation using homomorphic encryption, guaranteeing the security of genotype data when imputation is performed in a semi-honest environment. HE-based methods have comparable or lower time and memory requirements than non-secure methods. However, none of the above methods support both additive homomorphism and order-preserving properties.

### Homomorphic encryptions and order-preserving encryptions

There are many types of research on fully homomorphic encryption (FHE) and order-preserving encryption (OPE) schemes [17–19], most homomorphic encryption schemes do not satisfy the order-preserving property. In 2012, Liu et al. [20] proposed an order-preserving index scheme using simple linear functions and random noise to protect plaintexts. This scheme supports database range queries. But when there are duplicates in plaintexts, it might become vulnerable. Based on this work, a nonlinear order-preserving index scheme was proposed [21] and security has been improved. However, the scheme does not support additive homomorphism. An order-preserving encryption scheme together with trapdoor information was proposed by Liu et al. [22], which supports the server to perform k-means clustering directly over the encrypted data. In 2016, Liu et al. [23] propose a new simple OPE model that hides data distribution and frequency using message space expansion and nonlinear space split. The state encryption scheme greatly improves the efficiency of the algorithm and reduces the storage space of the key and ciphertext, but it could leak some plaintext information. We expect to design a privacy protection scheme that satisfies both additive homomorphism and order-preserving characteristics and saves computing cost.

### Motivations and contributions

Our main motivation in this paper is to design a secure outsourcing computation framework for solving the DDP problem. Considering that the DDP data is collected with days or even months of biological experiments and contains critical business interests, the privacy of DDP data should be protected securely. Otherwise, the DDP data owners are reluctant to deploy the task of solving DDP over cloud. Therefore, we need to first design a proper encryption algorithm to keep the privacy of DDP data. To solve the DDP problem, even for the cloud server, addition and sorting are two basic operations, thus our encryption scheme should support additive homomorphism and order-preserving. Furthermore, as aforementioned, among existing DDP algorithms, the QIGA outperforms others. Therefore, our previous QIGA algorithm is integrated into the proposed outsourcing framework. The difference of QIGA in this paper lies in that: The cloud server has to run QIGA on encrypted DDP data, while in our previous work, the DDP data owner runs QIGA on plaintexts of DDP data. By doing so, the original DDP instances need not be disclosed. Last but not least, the output of the cloud servers is mapping sequences that need not be encrypted. In the whole process, both the DDP data owner nor the cloud server need not decrypt the encrypted DDP instances. Thus, the involved order-preserving homomorphic encryption scheme is tailored into an order-preserving homomorphic index (OPHI) scheme by removing the decryption algorithm. In summary, our main technique contributions include three aspects:

- We first propose a privacy-preserving outsourcing framework for handling the DDP problem by using a cloud server;
- Then, to enable the cloud server to solve the DDP instances over ciphertexts, an order-preserving homomorphic index scheme is tailored from an order-preserving encryption scheme published at CCS 2012;
- Finally, our previous work on solving the DDP problem, a quantum inspired genetic algorithm (QIGA), is merged into our outsourcing framework, with the support of the proposed OPHI scheme.

## Results

We propose a security outsourcing computation framework PP-DDP for solving the double digest problem. Firstly, the data owner uses the proposed OPHI scheme to protect the privacy of the DDP instances, and then sends the encrypted data to the cloud server, which uses the QIGA algorithm to implement DDP calculation based on ciphertexts. Therefore, we carry out experimental analysis from three aspects: performance of OPHI scheme, performance of PP-DDP framework, and effects of privacy-preserving operations on the performance.

### Performance of OPHI scheme

In the PP-DDP framework, privacy protection mainly depends on the order-preserving homomorphic index scheme. OPHI scheme mainly has four functional modules: key generation, encryption, additive homomorphism and permutation operators. We test these four functional modules and each module run 1000 times to obtain the average running time. From Table 1, we can see that the average running time of each module is within $4\mu s$, the computing cost of these four modules reached microseconds and have good performance with little impact on DDP calculation.

**Table 1:** Module test of order-preserving homomorphic index scheme

Function module	Running time ($\mu s$)
Key generation *KeyGen*(*n*)	2.27
Encryption algorithm $Enc_k(m)$	2.42
Additive homomorphism	1.3
Permutation operator	3.8

### Performance of PP-DDP framework

**Table 2:** PP-DDP’s running result of typical instances

No.	Running time (*ms*)	Evolution generations	Success rate (%)
1	8.87	3.27	100
2	87.88	39.02	100
3	2.43	1.19	100
4	34.42	13.34	100
5	1.95	1	100
6	–	–	–
7	6.12	2.27	100
8	1.94	1.07	100

**Fig. 1:**
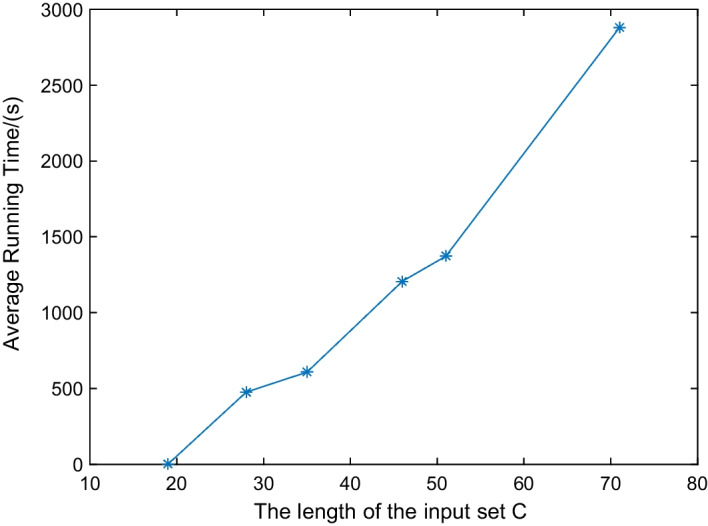
PP-DDP’s average running time of random instances. The length of the input set *C* ranges from 10 to 80. Running each instance 100 times

In order to verify the effectiveness and evaluate the performance of PP-DDP for the DDP problem, we selected eight typical instances in [6, 8], and randomly generated six sets of random instances to test PP-DDP respectively, then evaluated the performance of PP-DDP by average running time and average success rate. We randomly generated another six groups of random instances, and ran PP-DDP and QIGA at the same time for comparison experiments. In these experiments, we set the number of sub-ciphertext is $m=3$, the population size is $N=50$, the maximum evolutionary generation is 10000, the crossover probability is $pc=0.85$, and the mutation probability is 0.45–0.55. Running each instance 100 times to get the average running time and the average success rate. The goal of these experiments is to evaluate the feasibility of using PP-DDP to solve the DDP problem and the influence of privacy protection of the input instances on experimental results.

**Fig. 2:**
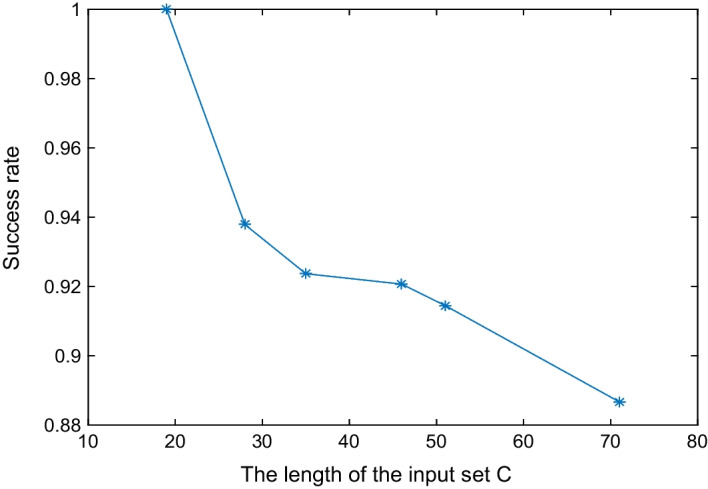
PP-DDP’s average success rate of random instances. The length of the input set *C* ranges from 10 to 80. Running each instance 100 times

We evaluated the effectiveness of PP-DDP by test the eight typical instances in [6, 8], the corresponding average running time and success rate are listed in Table 2. Instance 6 is an invalid instance which doesn’t satisfy $\sum _{i=i}^{m} a_{i}= \sum _{i=i}^{n} b_{i}= \sum _{i=i}^{k} c_{i}$. The success rate of the other seven instances is $100\%$, and all the average running times are within 0.088 s. PP-DDP efficiently solves these typical instances. Then, we generated six sets of random instances with the length of the input set *C* ranging from 10 to 80. The experimental results are in Figs. 1 and 2. It can be seen from Fig. 1 that the running time increases as the length of the set *C* increases, and in Fig. 2, the success rate decreases as the length of the set *C* increases. So, the length of the set *C* influences the experiment results, the larger the length of the set *C* is, the longer the running time and the lower the success rate. In Fig. 2, all success rates are above $88\%$, the PP-DDP is effective for these random instances.

Therefore, the proposed PP-DDP framework is effective for the DDP problem.

**Fig. 3:**
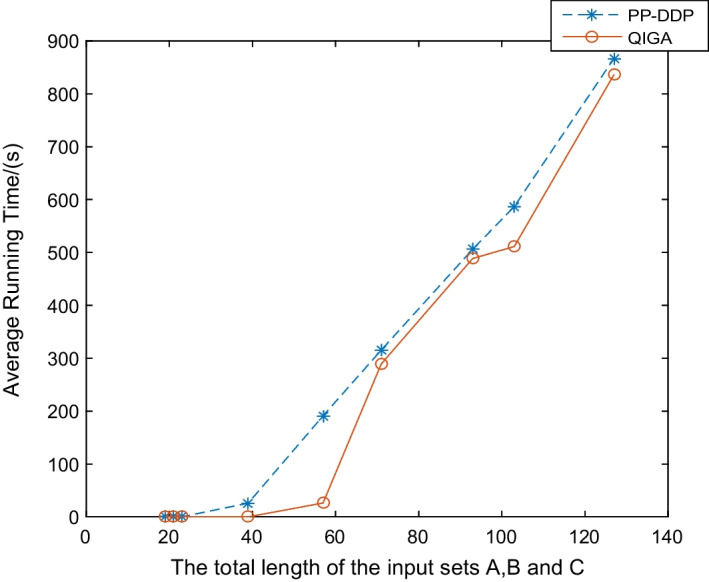
Comparison of the average running time of PP-DDP and QIGA for random instances. Running each random instance 100 times

**Fig. 4:**
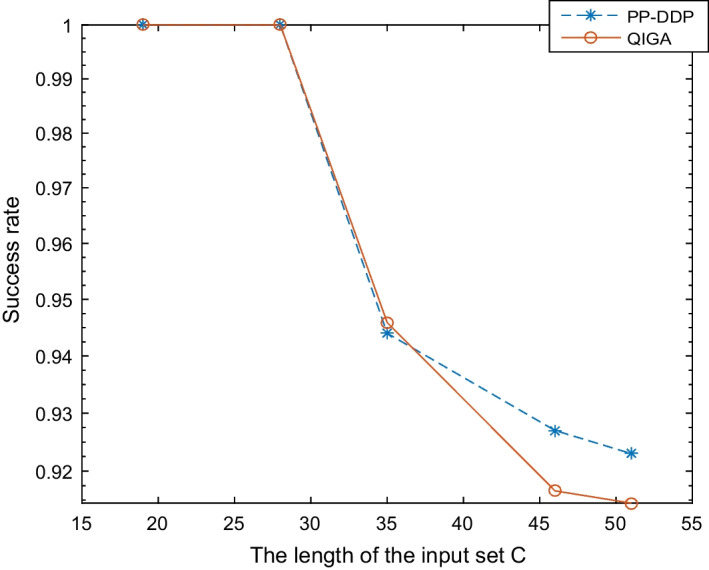
Comparison of the average success rate of PP-DDP and QIGA for random instances. Running each random instance 100 times

**Table 3:** Time cost of encryption

The total length of the input sets A, B and C	The percent of time cost (%)
19	68.83
21	65.58
23	80.42
39	99.87
57	86.28
71	8.28
93	3.46
103	12.87
127	3.53

### Effects of privacy-preserving operations on the performance

In sequel, let us give a simple analysis on the effects of privacy-preserving operations on the performance of the whole framework. We assess the effects based on the experimental results. PP-DDP and QIGA are compared by testing random instances. The difference between PP-DDP and QIGA is that PP-DDP provides privacy protection for input instances while QIGA does not. Figure 3 shows that the average running time of PP-DDP is slightly more than that of QIGA. We calculated the proportion of the time used by the privacy protection module to the total running time.

As shown in Table 3, when the number of input instance fragments is small, there will be 60–100% of the time cost to protect data privacy, when the input instance fragments are large, no more than $13\%$ of the time will be consumed. This is because, when the number of fragments is small, its own DDP experiment is microsecond, so the microsecond level of privacy protection module in the overall time accounted for a large proportion. When the number of fragments increases, the time required for the DDP experiment increases, but the time of the privacy protection module increases slowly, so the proportion in the overall time becomes smaller. Thus, the *greater* the number of the input DDP fragments, the *less* effects of privacy-preserving operations on the performance.

We can also see from Fig. 4 that the success rates of PP-DDP and QIGA are both above $90\%$. The difference between the success rate of PP-DDP and QIGA is very small, about 1%. Thus, the privacy protection module has little effect on the success rate of solving the DDP problem.

In brief, PP-DDP spent no more than $13\%$ of computational cost to achieve privacy-preserving on the DDP data at large volumes, and the privacy protection module has almost no effect on the success rate.

## Discussion

### Security analysis

In this section, we analyze the security of the PP-DDP framework in detail and prove that the framework can effectively resist the attacks proposed in Adversary model section.

**Fig. 5:**
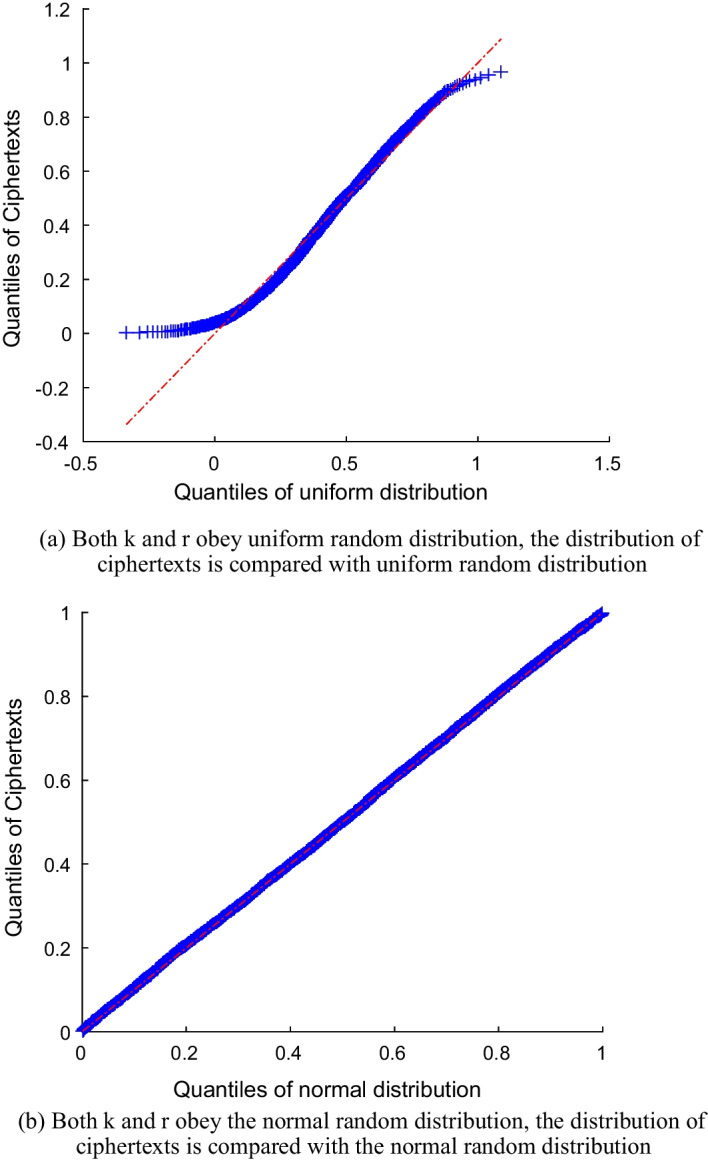
(In)distinguishable between ciphertext distributions and random distributions

- The honest but curious (semi-honest) cloud server: In this work, a secure outsourcing computation framework PP-DDP was proposed to solve the DDP problem. In the first stage of this framework, the input plaintext DDP data is encrypted through the order-preserving homomorphic index scheme. The proposed order-preserving homomorphic index scheme is a symmetric encryption scheme. As the plaintexts, keys and encryption mechanism are not disclosed to the cloud servers, who can only obtain ciphertexts and the output mapping sequences. It is required that the cloud servers cannot decrypt the ciphertexts from the existing public parameters, so the scheme should satisfy one-wayness security.
  The proof of one-wayness is as follows: from the OPHI scheme, $0<k_im<c_i<k_i(m+p)$, so the ciphertext space will expand $k_i$ times after encryption, and the probability of obtaining the correct plaintext through exhaustive search is $\frac{1}{k\mathcal {M}}<\frac{1}{2^{17}}$, and there are $m+n+k$ plaintexts in the DDP problem, where *m*, *n*, *k* are the lengths of the input sets *A*, *B*, *C* respectively, $m+n+k\ge 7$, so the probability of the adversary getting the solution of the DDP problem is $(\frac{1}{ k\mathcal {M}})^{m+n +k}<\frac{1}{2^{110}}$, which is apparently negligible. Therefore, the OPHI scheme satisfies one-wayness.
  In the PP-DDP framework, the cloud servers will honestly implement the requirements of the data owners and send a pair of mapping sequences of the DDP calculation to the data owners. The mapping sequences are only the arrangement of the index and can’t reflect any information in the plaintexts. The mapping sequences don’t need to be encrypted during the sending process, and can be completely disclosed. Therefore, the cloud servers can’t recover the plaintexts from the mapping sequences.
- The lazy cloud server: The cloud servers return a pair of mapping sequences to the data owners. If the mapping sequences are generated randomly, the correct probability is only $\frac{1}{A_{m}^{m}A_{n}^{n}}$, the data owners can verify whether the result is correct through simple calculation, as shown in Modeling the DDP problem section. Once the result is wrong, the data owners will not use the result and ask the cloud servers to recalculate until the correct result is obtained.
- The malicious competitors of the DDP data owner: The original DDP data has commercial value which was obtained by gel-electrophoresis experiments cost a lot of time and money. Malicious competitors want to get the original DDP data. However, only encrypted DDP data and DDP solution are transmitted over the public channel. So its security analysis is the same as “The honest but curious (semi-honest) cloud server”.
- We also analyze the security of the OPHI scheme through experiments. Quantile-Quantile plot (QQ plot) is a graphical method that compares two probability distributions by drawing quantiles. If the two distributions being compared are similar, the QQ graph is approximately located on the line $y=x$. In the order-preserving homomorphic index scheme, $c_i=k_i\times m+r_i$, where *m* is an integer in the plaintext space, and *k* and *r* are random numbers. We conduct two sets of experiments to verify whether the ciphertexts are consistent with the uniform distribution or normal distribution.
  In Fig. 5a, we randomly generate 50, 000 keys *k* and noise *r* satisfying the uniform random distribution. Compared with a uniform random distribution. The points of the generated ciphertexts in the QQ plot are close to the line $y=x$ with a little deviation, therefore, when key *k* and noise *r* are subject to the uniform distribution, the ciphertexts generated by this scheme are close to the uniform random distribution, but a certain amount of information will be leaked.
  In Fig. 5b, we randomly generate 50, 000 keys *k* and noise *r* are subject to the normal random distribution. While comparing the generated ciphertexts with the normal random distribution, in Fig. 5b, the points coincide with the line $y=x$, indicating that when both the keys and noise subject to the normal random distribution, the distribution of ciphertexts is indistinguishable from the normal distribution. Therefore, in Order-preserving homomorphic index scheme section, the generated key *k* and noise *r* both satisfy the normal distribution.

### Complexity analysis

The OPHI scheme is proposed to protect the privacy of the DDP instances. In this scheme, the plaintext is encrypted into a ciphertext vector that contains *n* sub-ciphertexts. For given a DDP instance (*A*, *B*, *C*), let $L=|A|+|B|+|C|$, i.e. the total number of elements in three sets *A*, *B*, *C*, then the time complexity of privacy-preserving operations, i.e. the execution of OPHI scheme, is $\mathcal {O}(n\cdot L)$.

Upon receiving these encrypted DDP instance from the DDP data owner, the cloud server executes QIGA based on ciphertexts. QIGA mainly contains five steps, the complexity of each step is given below:

- Step 1: Initialize the population with the complexity of $\mathcal {O}(N\cdot L)$, where *N* is the population size.
- Step 2: Measure every individual, the total complexity is $\mathcal {O}(N\cdot L\log L)$.
- Step 3: Evaluation of each individual with the total complexity of $\mathcal {O}(g\cdot L\cdot N)$. Where *g* is the maximum evolution generation.
- Step 4: The quantum selection operator is used to determine whether the termination condition is satisfied, the complexity is $\mathcal {O}(g\cdot N)$.
- Step 5: Update the population. Perform quantum rotation gate, quantum crossover and quantum mutation operations sequentially, the total complexity is $\mathcal {O}(g\cdot L\cdot N)$. (Note that in practice, the running time would be observably less since all these genetic operators are performed according to the setting probability. In the sense of complexity, we neglect these constants of course.)

Thus, the total complexity of QIGA *by classical simulation* is

$$\begin{aligned}{} & {} \mathcal {O}(N\cdot L)+\mathcal {O}(N\cdot L\log L)+\mathcal {O}(g\cdot L\cdot N)+\mathcal {O}(g\cdot N)\\{} & {} \quad +\;\mathcal {O}(g\cdot L\cdot N)=\mathcal {O}(g\cdot L\cdot N\cdot \log L). \end{aligned}$$

While in the future quantum computation era, when fully fledged quantum computers are available, our quantum inspired genetic algorithm should be adapted to a real quantum settings, i.e. quantum genetic algorithm with the quantum complexity $\mathcal {O}(g\cdot L\log L)$, since at that time all *N* chromosomes would be represented by a single chromosome in a quantum superposition state.

Last but not the least important is that in the sense of asymptotically, the prvivacy-preserving operations have no effects on the complexity of the whole system, since the complexity term $\mathcal {O}(n\cdot L)$ would be absorbed totally by the term $\mathcal {O}(g\cdot L\cdot N\cdot \log L)$ considering that $n\ll N$ holds in general.

### Comparison with other algorithms for the DDP problem

**Fig. 6:**
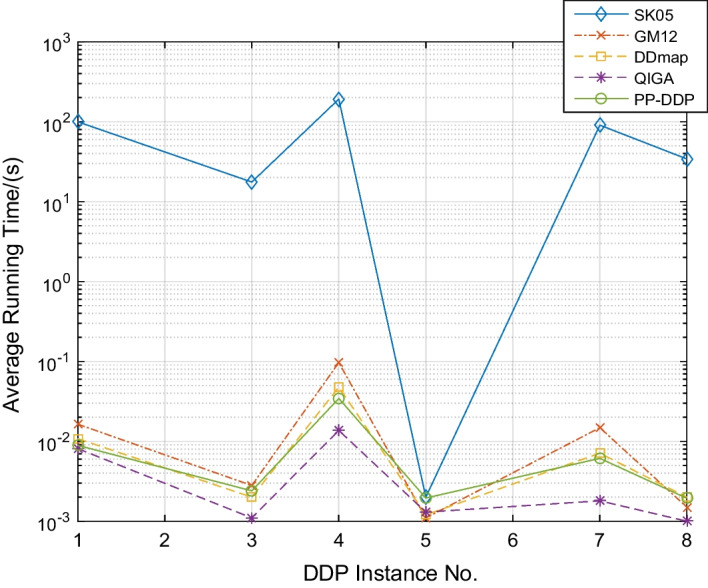
Comparison of the average running time of PP-DDP and the other four algorithms for instances in [6, 8]. DDmap is the best performance operator in [8], SK05 is the genetic operator in [6] and GM12 is the genetic operator in [7], QIGA is the operator in [9]

The proposed PP-DDP framework compare with the other four algorithms (SK05 [6], GM12 [7], DDmap [8] and QIGA [11]) for solving the DDP problem. Running instance 1, 3, 4, 5, 7, 8 in [6, 8] 100 times. It can be seen from Fig. 6 that the line of SK05 is at the top, SK05 asks for a running time much longer than the other four algorithms, which performs the poorest. There is only a slightly difference among the other four algorithms, and the comparison of their running time is as follow: QIGA<PP-DDP<DDmap<GM12<SK05, QIGA requires the shortest running time, PP-DDP is the second, yet PP-DDP protects the privacy of the DDP data during the whole process and the third party cannot obtain the DDP data-related information. However, data privacy-preserving is not taken into account in QIGA. Malicious third parties may steal these data to obtain genetic information. In summary, PP-DDP improves data security and protects genetic information at the expense of a very small running time difference.

### Comparison with other privacy-preserving techniques

In the proposed PP-DDP outsourcing computation framework, the privacy-preserving technology of data owner must satisfy both additive homomorphism and order-preserving property. Most of the existing privacy-preserving mechanisms are not suitable for our scenario. Our core motivation is to design a secure outsourcing computation framework to solve the DDP problem. As far as we know, this is the first secure outsourcing computation framework for the DDP problem. Furthermore, considering that the most complex workload in the DDP scenario is the QIGA rather than the OPHI process. Therefore, we only compare PP-DDP with existing algorithms for solving DDP problems in the aspect of computational overhead.

In terms of functions, we compare PP-DDP with existing privacy-preserving technologies, including secure multi-party computation (SMPC), differential privacy (DP), homomorphic encryption (HE), and order-preserving encryption (OPE). We present comparisons among our PP-DDP proposal and these technologies from the following aspects: additive homomorphism, order-preserving, computational cost, and DDP applicability. The results are given in Table 4. We can see that none of the existing techniques is applicable to the DDP problem.

**Table 4:** Function comparison of privacy protection technologies

Privacy-preserving technologies	SMPC	DP	HE	OPE	PP-DDP
Additive homomorphism	Yes	No	Yes	No	Yes
Order-preserving	No	No	No	Yes	Yes
Computational cost	High	Low	High	Low	Low
DDP applicability	No	No	No	No	Yes

## Conclusion

To solve the double digest problem (DDP) effectively by resorting the power of cloud computation and meanwhile protecting the business interests of the DDP data owner, a privacy-preserving outsourcing framework is proposed in this work. This framework mainly consists of two parts, OPHI and QIGA. The former is executed by the DDP data owner, while the latter is deployed on the cloud server. OPHI encrypts the input instances and supports additive homomorphism and order-preserving properties, while QIGA finds the optimal solutions, i.e. mapping sequences, for the encrypted DDP instances. Our experiments show that on one hand, for both the public test DDP instances and the random DDP instances, the success rate of our proposal is above $88\%$, on the other hand, the proposed framework takes no more than 13% of computational cost to achieve privacy-preserving functionality on the DDP data, and has almost no impact on the success rate. In the future of the quantum computation era, it is also interesting to replace the QIGA part with a fully-fledged quantum genetic algorithm, for obtaining an even high success rate in solving even large DDP instances.

## Methods

### Overview of system

In this section, we briefly discuss the system model, adversary model, and design objective.

#### System model

**Fig. 7:**
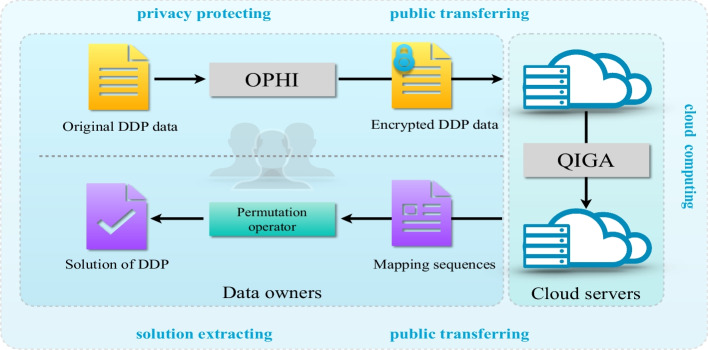
The flowchart of PP-DDP. Step 1: privacy protecting. Step 2: public transferring. Step 3: cloud computing. Step 4: public transferring. Step 5: solution extracting. OPHI is the order-preserving homomorphic index scheme, and QIGA is the quantum inspired genetic algorithm

In this work, a secure outsourcing computation framework for the DDP problem has been proposed, named PP-DDP. As shown in Fig. 7, this system includes 2 entities, data owners and the cloud servers.

- Data owner (DO): The data owner has the ability to store and simple computing data. It has a large amount of DDP data obtained through electrophoresis experiments. However, due to its limited computing resources, the calculation of the DDP data should be outsourced to cloud servers. The data owner uses the order-preserving homomorphic index scheme to encrypt the DDP data that he owns, and then sends the encrypted ciphertext DDP data to the cloud servers.
- Cloud server (CS): It is a service provider that provides cloud computation services, allowing paying customers to use powerful computing resources for data computing, helping customers reduce costs. However, it must be “honest but curious”, i.e. it will honestly execute certain algorithms, but is interested in the user’s private data.

Based on the system model, we define the order-preserving homomorphic index scheme as follows:

##### Define 1

Order-preserving homomorphic index scheme (OPHI) is an encryption scheme that satisfies the order-preserving and homomorphic properties, it doesn’t require data decryption. This scheme consists of the following two algorithms:

- $KeyGen(n)\rightarrow \overrightarrow{K}$ is a key generation algorithm run by the data owner. It outputs a *n*-dimensional key vector $\overrightarrow{K}$.
- $Enc(\overrightarrow{K},m)\rightarrow \overrightarrow{c}$ is a encryption algorithm. It takes $\overrightarrow{K}$ and a plaintext *m*, and outputs the *n*-dimensional ciphertext vector $\overrightarrow{c}$.

#### Adversary model

For our proposed PP-DDP framework, we consider the following three adversary models:

- The honest but curious (semi-honest) cloud server that might want to recover the original DDP data. We limit cloud servers attack to passive attacks. It is assumed that the cloud servers honestly perform certain computations on encrypted data and send complete and correct results. Yet cloud servers are curious and may try to obtain information about plaintext DDP data from ciphertext data and mapping sequences.
- The lazy cloud server that might return random results. The cloud server may only charge users, but don’t perform specific computations. They may randomly generate a pair of mapping sequences and send them to the data owners.
- The competitors of the DDP data owner that want to learn both the original DDP data and the DDP solution. DDP data has commercial value, for commercial purposes, competitors may steal data from public channels. The data on public channels include the encrypted DDP data by the data owners and the mapping sequences returned by the cloud servers.

#### Design objective

In order to realize the secure outsourcing computation of the DDP problem, the core is that the cloud servers cannot infer the DDP data which has commercial value, we have the following design goals:

- Data confidentiality: The cloud servers shouldn’t be able to recover any useful information from any encrypted data, ensuring that the cloud servers can’t access sensitive data.
- Index privacy: The proposed index results, the two mapping sequences can’t reveal information about the original plaintexts.
- Efficiency: Since real-time results may not be required for DDP experiments, computation time in the cloud is tolerable to a certain extent. However, the computation of the data owners must be limited because their computing resources are usually limited.

### Modeling the DDP problem

The double digest problem is a problem in constructing physical maps of DNA sequences. Digest experiment can be described as below: An enzyme cuts a DNA sequence at specific positions. Different enzymes cut DNA sequences at different restriction cleavage sites. Now two kinds of enzymes $\alpha$ and $\beta$ are used to cut the same DNA sequence in three ways: firstly, using enzymes $\alpha$; secondly, using enzymes $\beta$; thirdly, using them simultaneously. Then, we can obtain three multisets *A*, *B*, *C* of the length of DNA fragments, we call them DDP instances. In this work, we first encrypt the DDP instances, and then, use the quantum inspired genetic algorithm to reorder these encrypted DNA fragments to find the optimal mapping sequences $\mu$ and $\nu$. The mathematical description of the DDP problem is as follows:

$A=\{ a_{1}, a_{2},\cdots , a_{p} \}$, $B=\{b_{1}, b_{2},\cdots , b_{q}\}$, and $C=\{c_{1}, c_{2},\cdots , c_{t}\}$ are input DDP instances in ascending order, they satisfy $\sum _{i=i}^{p} a_{i}= \sum _{i=i}^{q} b_{i}= \sum _{i=i}^{t} c_{i}$, The encrypted instances become $A_c=\{ (a_{11}, a_{12},\cdots , a_{1j}),\cdots ,(a_{p1}, a_{p2},\cdots , a_{pj}) \}$, $B_c=\{ (b_{11}, b_{12},\cdots , b_{1j}),\cdots ,(b_{q1}, b_{q2},\cdots , b_{qj}) \}$, $C_c=\{ (c_{11}, c_{12},\cdots , c_{1j}),\cdots ,(c_{t1}, c_{t2},\cdots , c_{tj}) \}$, where *p*, *q* and *t* are lengths of the sets *A*, *B* and *C*, respectively. There have two mapping sequences $\mu$ and $\nu$ which are the permutations of the indices $[1,2,\cdots ,p]$ and $[1,2,\cdots ,q]$ respectively. After mapping by $\mu$ and $\nu$, $\overrightarrow{A_{c\mu }}=[ A_{1},A_{2},\cdots ,A_{p} ]$, $\overrightarrow{B_{c\nu }}=[ B_{1},B_{2},\cdots ,B_{q} ]$ can be obtained.

#### Define 2

$\overrightarrow{AS(A)}$ is *accumulative summation* of *A* and *step*  *difference* of *A* denote as $\overrightarrow{SD(A)}$,

$$\begin{aligned} \overrightarrow{AS(A)}= & {} \left[ \sum _{i=1}^{1}a_{i},\sum _{i=1}^{2}a_{i},\cdots ,\sum _{i=1}^{p}a_{i}\right] ,\\ \overrightarrow{SD(A)}= & {} \left[ \sum _{i=1}^{2}a_{i}-\sum _{i=1}^{1}a_{i},\cdots ,\sum _{i=1}^{p}a_{i}-\sum _{i=1}^{p-1}a_{i}\right] \end{aligned}$$

Accumulative summation and step difference of $\overrightarrow{A_{c\mu }}$ and $\overrightarrow{B_{c\nu }}$ yields $\overrightarrow{C_{\mu ,\nu }}=[ C_{1},C_{2},\cdots ,C_{t} ]$. Rewrite $\overrightarrow{C_{\mu ,\nu }}$ in increasing order $\overrightarrow{C'_{\mu ,\nu }}=[C'_{1},C'_{2},\cdots ,C'_{t}]$.

The objective of the DDP problem is to find two optimal mapping sequences $\mu$ and $\nu$ which satisfy the condition of $C'_{\mu ,\nu }=C_c$, considering the existence of the partial cleavage error, the optimization goal is updated to $min \sum |C'_{\mu ,\nu }- C_c|$.

### Outsourcing computation framework for the double digest problem

This work proposes an outsourcing computation framework for the double digest problem, which aims to solve the DDP problem under the premise of protecting the privacy of genetic data. The advancement of cloud computing technology makes large scale computation more affordable than before, as the data owners can outsource their massive computing tasks to cloud servers to save cost. Similarly, due to the large scale of data and high complexity of calculation in the double digest experiments, we chose to use the PP-DDP outsourcing computation framework. In this framework, the first step is to protect the privacy of the length of the DNA fragments obtained in the double digest experiments, which uses the order-preserving homomorphic index scheme to encrypt the data to protect the privacy. The attacker can only compare the length of these fragments, but cannot obtain the specific value. Then send the encrypted DDP data to the cloud servers. The cloud servers process the encrypted data and use the quantum inspired genetic algorithm to obtain the solution to the DDP problem. It is unnecessary to decrypt the data in the model, the output is mapping sequences. The attacker can not get the DDP data even if the output result is obtained.

In the PP-DDP framework, the whole business logic is divided into 5 stages (Fig. 7):

- The first stage is to protect the privacy of the DDP instances, by using the proposed order-preserving homomorphic index (OPHI) scheme. That is the original DDP data is encrypted.
- The second stage is to transfer these encrypted DDP instances from the data owners to the cloud server. The adversary, even the cloud server, cannot break the privacy of these DDP instances.
- The third stage is the most complex process of the whole work, i.e. our quantum inspired genetic algorithm (QIGA). This is purely a cloud computing process, handled by the cloud server. Since the DDP instances are encrypted, this process is finished based on ciphertexts, owing to the capability of the proposed OPHI scheme that supports additive homomorphism and order-preserving property over ciphertexts.
- In the fourth stage, the cloud server transfers the “solution” to the data owner via a public channel. Note that the adversary can learn nothing from this publicly transferred “solution”, since without knowing the original DDP data, this “solution” is merely mapping sequences based on encrypted DDP instances.
- In the fifth stage, the data owner extracts the real solution by combing the mapping sequences with the original DDP instances.

#### Remark 1

(Why combine order-preserving encryption with homomorphic encryption) Quantum inspired genetic algorithms have shown obvious advantages in solving DDP problems, so in step 3 of the outsourcing computation framework, quantum inspired genetic algorithms are used for DDP computation. In Security analysis section, we can see accumulative summation and step difference operator contain additive operation and subtraction operation, from $\overrightarrow{C_{\mu ,\nu }}$ to $\overrightarrow{C'_{\mu ,\nu }}$, there have sorting operation, so the encryption scheme in step 1 must satisfy both additive homomorphism and order-preserving properties. Therefore, it’s necessary to combine order-preserving encryption with homomorphic encryption to propose an order-preserving homomorphic index scheme for the DDP problem.

### Order-preserving homomorphic index scheme

Homomorphic encryption is an encryption method that the algebraic operation on the plaintext during encryption is equivalent to another operation performed on the ciphertext. The encryption function of two plaintext *a* and *b* satisfy $Dec(Enc(a)\odot Enc(b))=a \oplus b$, where *Enc* is the encryption operation, *Dec* is the decryption operation, $\odot$ and $\oplus$ respectively correspond to the operations in the plaintext and ciphertext fields. In recent years, cloud computing has attracted lots of attention, one of the problems encountered in practical applications is how to guarantee data security, which can be solved by homomorphic encryption with the feature mentioned above.

In this work, the encryption scheme must satisfy both additive homomorphism and order-preserving properties. There have been already several schemes for homomorphic encryption and order-preserving encryption. In 1978, Rivest et al. [24] firstly came up with the concept of homomorphic encryption. In the beginning, the proposed schemes were all partial homomorphic encryption (PHE) schemes, that is, they supported either homomorphic addition or multiplication. Typical PHE schemes include the schemes of ElGamal [25], Paillier [26], etc. Until 2009, Gentry [17] put forward the first fully homomorphic encryption (FHE) scheme based on the ideal lattice. After that, many researchers have improved Gentry’s scheme, for example [27–29]. In addition, there have been some FHE schemes based on integer and LWE [30, 31].

Order-preserving encryption (OPE) was first proposed by Agrawal et al. [32] in 2004, but they did not provide formal security proof. In 2009, Boldyreva et al. [33] put forward an order-preserving encryption scheme based on search trees and gave formal security proof. In 2013, Popa RA et al. [34] proposed an ideal security mutable order-preserving encoding (mOPE) model. Despite these advances, most OPE schemes don’t support homomorphic operators and they are deterministic schemes, which can neither reach semantic security nor resist frequent attacks. Thus, they cannot solve the DDP problem. In 2012, Liu et al. [20] came up with an order preserving indexing scheme, using simple linear functions and random noise to protect plaintexts. Based on this work, they proposed a nonlinear indexing scheme to address the vulnerability of linear indexing [21]. In 2014, Liu et al. [22] proposed an identity that does not require a noise reduction mechanism. The state encryption scheme greatly improves the efficiency of the algorithm and reduces the storage space of the key and ciphertext, but it could leak some plaintext information. Liu’s scheme is a probabilistic scheme, which is one-way security and does meet our requirements. So we choose Liu’s scheme.

Based on the scheme of Liu et al. [20, 21], we propose an order-preserving homomorphic index scheme.

Order-preserving homomorphic index scheme (OPHI) needs to meet the following conditions:

- OPHI is an encryption scheme, it can protect data privacy;
- OPHI supports additive homomorphism operation, $Enc(m_1)+Enc(m_2)=Enc(m_1+m_2)$;
- OPHI supports order-preserving index, for any plaintext $m_1$ and $m_2$, when $m_1>m_2$, there are $Enc(m_1)>Enc(m_2)$, iff $c_{1j}>c_{2j},j=1,2,\cdots ,n-1$.

The basic idea of OPHI is the plaintext *m* becomes a ciphertext vector containing *n* sub-ciphertexts after encryption. $Enc_k(m)=[c_1,\cdots ,c_n]$. The encryption process can be presented as:

- Key generation algorithm *KeyGen*(*n*):
  Generate a key vector containing *n* real numbers $\overrightarrow{K}=[k_1,\cdots ,k_n]$, which subjects to the normal distribution and the following restrictions,
  $$\left\{ {\begin{array}{*{20}l} {k_{1} + k_{2} + \ldots + k_{{n - 1}} \ne 0} \hfill \\ {0 < k_{i} \le 2^{{10}} } \hfill\\\end{array} } \right.$$
- Encryption algorithm $$Enc_k(m)$$:
  Given plaintext $m\in \mathcal {M}$ and key $\overrightarrow{K}$, it generates a noise vector containing $n-1$ random numbers and satisfies the normal distribution, $\overrightarrow{r}=[r_1,\cdots ,r_{n-1}]$, let $R=r_1+r_2+\cdots +r_{n-1}$ and $0<R<k_ip$, *p* is the minimized difference between any two plaintexts, $p=min |m_1-m_2|$. The sub-ciphertexts are
  $$c_{i} = \left\{ {\begin{array}{*{20}l} {k_{i} \times m + r_{i} } \hfill & {\left( {1 \le i \le n - 1} \right)} \hfill \\ {k_{n} \times \left( {r_{1} + \cdots + r_{{n - 1}} } \right)} \hfill & {\left( {i = n} \right)} \hfill \\ \end{array} } \right.$$

- Homomorphism: This scheme is a homomorphic index scheme, which satisfies additive homomorphism, the detailed proof process is presented as below:
  For plaintext $m_1$ and $m_2$, the corresponding ciphertexts are
  $$\begin{aligned} C_1= & {} Enc_k(m_1)=[c_{11},\cdots ,c_{1j},\cdots ,c_{1n}]\\ C_2= & {} Enc_k(m_2)=[c_{21},\cdots ,c_{2j},\cdots ,c_{2n}] \end{aligned}$$
  when $$1\le j\le n-1$$,
  $$\begin{aligned} \begin{aligned} Enc_k(m_{1j})+Enc_k(m_{2j})&=k_im_1+r_1+ k_im_2+r_2\\&=k_i(m_1+m_2)+r3\\&=Enc_k(m_{1j}+m_{2j}) \end{aligned} \end{aligned}$$
  when $$j=n$$, $$R=r_1+\cdots +r_{n-1}$$,
  $$\begin{aligned} \begin{aligned} Enc_k(m_{1n})+Enc_k(m_{2n})&=k_nR_1+k_nR_2\\&=k_nR_3\\&=Enc_k(m_{1n}+m_{2n}) \end{aligned} \end{aligned}$$
  Therefore, this is an additive homomorphism index scheme.
- Order-preserving: The proof of order-preserving property is as follows:
  when $$0<j\le n-1$$,
  $$\begin{aligned} \begin{aligned} c_{1j}-c_{2j}=&\;(k_im_1+r_1)-(k_im_2+r_2)\\ =&\;k_i(m_1-m_2)+(r_1-r_2) \end{aligned} \end{aligned}$$
  since $0<R<k_i\times \textrm{min}|m_1-m_2|$, then $0<r_i<k_i\times \textrm{min}|m_1-m_2|$, therefore $-k_i\times \textrm{min}|m_1-m_2|<r_1-r_2<k_i\times \textrm{min}|m_1-m_2|$, when $m_1>m_2$, $m_1-m_2>0$, we deduce that $c_{1i}-c_{2i}>0$, thus this scheme satisfies the order-preserving character. When $j=n$, sub-ciphertext $c_{in}$ has nothing to do with plaintext.

In conclusion, this is an order-preserving homomorphic index scheme.

### Quantum inspired genetic algorithm

**Fig. 8:**
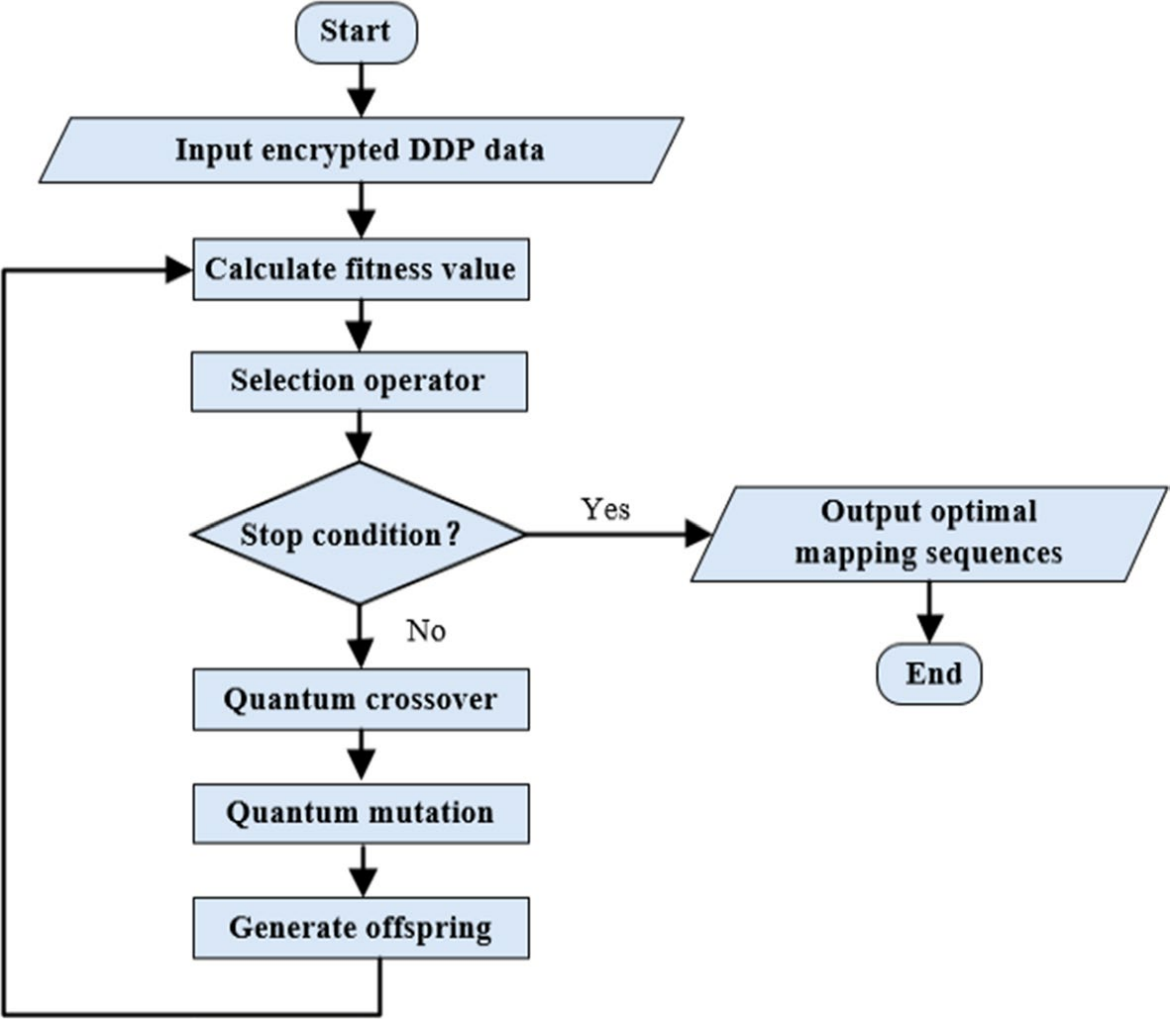
The flowchart of QIGA. The input instances are the ciphertexts encrypted by the order-preserving homomorphic index scheme, after calculating the fitness value, if not satisfied with the stop condition, the quantum crossover and quantum mutation operators will be performed and generated new offsprings. Otherwise, output the optimal results

Genetic algorithm (GA) is designed and proposed according to the evolutionary laws of organisms in nature. It is a calculation model of the biological evolution process that simulates the natural selection and genetic mechanism of Darwin’s biological evolution theory to search for the optimal solutions. Quantum computing has the ability of parallel computing. In order to improve the capability of classical genetic algorithms to solve the DDP problem, researchers combined genetic algorithms and quantum computing to propose quantum inspired genetic algorithms (QIGA). When solving more complex combinatorial optimization problems, researchers can usually obtain the optimization results in a shorter time with quantum inspired genetic algorithms, compared with some conventional optimization algorithms.

In 2020, Suo et al. [11] proposed a quantum inspired genetic algorithm to deal with the DDP problem, and QIGA slightly accelerates to solve the problem. In this work, we firstly use the order-preserving homomorphic index scheme to encrypt the DDP instances and then use the quantum inspired genetic algorithm in [10] to calculate the encrypted instance to find the optimal mapping sequences. The difference between the proposed quantum inspired genetic algorithm and the QIGA in [11] is that the fitness function is different. In this work, the fitness function is $f(\mu ,\nu )=\frac{1}{1+|C'_{\mu ,\nu }\oplus C_c|}$. $C'_{\mu ,\nu }$ is the reordered sequence $C'$ obtained through the PP-DDP framework, and $C_c$ is the encrypted sequence obtained through the order-preserving homomorphic index scheme. The optimal fitness value is 1, and the closer the fitness value is to 1, the closer the solution to the optimal solution. The algorithm flow chart is described in Fig. 8.

## Data Availability

PP-DDP is open source and available at https://github.com/ronghaoZHI/PPQIGA.

## Abbreviations

DDP: Double digest problem
QIGA: Quantum inspired genetic algorithm
OPHI: Order-preserving homomorphic index scheme
NGS: Next-generation sequencing technology
FHE: Fully homomorphic encryption
PHE: Partial homomorphic encryption
OPE: Order-preserving encryption
GA: Genetic algorithm
